# Implementing change for facility-based peripartum care in low-income and middle-income countries

**DOI:** 10.1016/S2214-109X(20)30306-5

**Published:** 2020-08-01

**Authors:** Jalemba Aluvaala, Mike English

**Affiliations:** Kenya Medical Research Institute-Wellcome Trust Research Programme, Nairobi, Kenya; Department of Paediatrics and Child Health, College of Health Sciences, University of Nairobi, Nairobi, Kenya; Kenya Medical Research Institute-Wellcome Trust Research Programme, Nairobi, Kenya; Centre for Tropical Medicine and Global Health, Nuffield Department of Medicine, University of Oxford, Oxford, UK

The quality gap in facility care for small and sick neonates in low-income and middle-income countries has to be bridged to achieve 12 or fewer deaths per 1000 livebirths.^1^ Dilys Walker and colleagues^2^ did a cluster-randomised facility-based trial in Kenya and Uganda to evaluate the effect of a multifaceted intervention on fresh stillbirth and neonatal mortality among preterm and low-birthweight births. In the available literature, maternal mortality, stillbirths, and early neonatal mortality have been shown to be linked, but stillbirths are often not included in the studies even though fresh stillbirths are a key indicator of intrapartum care. By contrast, Walker and colleagues included stillbirths as a key target of the implemented package of care.^3^

The investigators strengthened data collection and introduced a modified Safe Childbirth Checklist in all 20 sites. The data collection strengthening included monthly data quality assurance visits, a data dashboard with monthly site reports, and bi-annual dissemination of findings with facility stakeholders. Ten intervention sites were involved in country-specific quality improvement collaboratives (with between 3–6 meetings) linked to onsite quality improvement teams that met every two weeks during the trial. Local mentors were used, and simulation and team training lasting 4 days, repeated every 5–6 weeks, were done in Kenya and simulation and team training lasting 2 days, repeated every 6–8 weeks, were done in Uganda.^4^ The study is an important contribution indicating what efforts are required to translate evidence-based recommendations into practice in settings with the largest burden of stillbirths and neonatal mortality,^5^ in populations that are still rarely included in large pragmatic trials.^6^ The authors showed a 34% reduction in the odds of the composite outcome of fresh stillbirth and neonatal mortality (0·66, 95% CI 0·54-0·81) in the intervention sites compared with the control sites.

The study offers many important lessons. First, it is illustrative that neither in Kenya nor in Uganda were routine data on stillbirth and even infacility neonatal deaths adequate for tracking such basic indicators of health system quality. Second, the study points to how hard service providers, policy makers, and researchers might have to work with rural facilities to have a large effect on outcomes, although, reassuringly, an effect seems possible without substantial investment in advanced technologies. Thirdly, the study poses questions of how such interventions can be implemented at a larger scale so that the interventions continue to support the acquisition of essential data and yield further improvements in care and outcomes. The authors report that measures of facility readiness were lower in both study arms at the end of their study than at its start. As part of this reporting, and in keeping with emerging good practice in implementation science, it would be useful to explore applying more theoretically informed frameworks to explain how the interventions changed behaviours and outcomes;^7^ such application would help others to replicate such intervention evaluations, as the authors recommend.

Finally, the study illustrates how difficult it is to do large intervention studies and how important it is to do careful reporting and cautious interpretation. One challenge is defining outcomes as differences in the reporting of outcomes in perinatal and neonatal care could preclude comparison across studies. Walker and colleagues^2^ reclassified some stillbirths as neonatal deaths, which were recorded as having a 1-min-after-birth Apgar score greater than zero. The development and implementation of core outcome sets to standardise reporting across studies in low-income and middle-income countries would be useful.^8^ These sets might include important outcomes beyond survival, such as early postnatal growth in preterm or low-birthweight survivors and other sick neonates during inpatient care in neonatal units. Another challenge is obtaining good data. In Walker and colleagues’ study, 15% of the key register data were missing and 15% (440/2882) of the babies eligible for the post-discharge 28-day outcome evaluation were lost to follow-up, despite the efforts of the study team. Other studies that used standardised routine information tools coupled with regular audit and feedback observed similar rates of missingness,^9^ but these problems can be partly addressed by using imputation strategies to avoid data loss. Walker and colleagues also acknowledge the potential for bias attributable to the baseline or emerging imbalance across the facilities that were assigned to the intervention and control groups, a challenge that is common when randomising complex organisations.^10^ Thus workloads, caesarean rates, and prevalence of low 5-min-after-birth Apgar scores were higher in control than intervention facilities. Although the 34% reduction in odds for fresh stillbirth and 28-day neonatal mortality among preterm or low-birthweight babies is exciting, there was a trend but no statistically significant effect of the intervention across all births; this analysis included approximately triple the number of adverse outcomes in total (1·8% [974/53 920] of fresh stillbirths and 1·1% [580/53 920] of pre-discharge neonatal deaths).

When considering further research, however, the global health community should not forget some of the fundamental bottlenecks in delivering peripartum care. Among the principal bottlenecks are the inadequate human resources for health and the deficits in the nursing workforce, who are the pillar of delivering facility-based reproductive, maternal, newborn, and child health services. The global health community should also not neglect the importance of effective referral systems.
